# Supplementary material for: Predicting visual function by interpreting a neuronal wiring diagram
Source: Nature. 2024 Oct 2;634(8032):113–23. doi: 10.1038/s41586-024-07953-5 (PMC11446822; doi:10.1038/s41586-024-07953-5)
Supplement: Supplementary file 6 — CRF and ERF predictions for individual TmY4 and TmY9 cells. Analogous to Supplementary Data 3, but for TmY target types. Shown are the top four monosynaptic pathways, the strongest pathway passing through each of the top ten intermediary types (ranking from Extended Data Fig. 7), and the trisynaptic pathway Tm1–TmY–Dm3–TmY (see the section entitled Prediction of spatial normalization). [file 41586_2024_7953_MOESM6_ESM.zip › DataS4/TmY9q/720575940639507790.pdf]

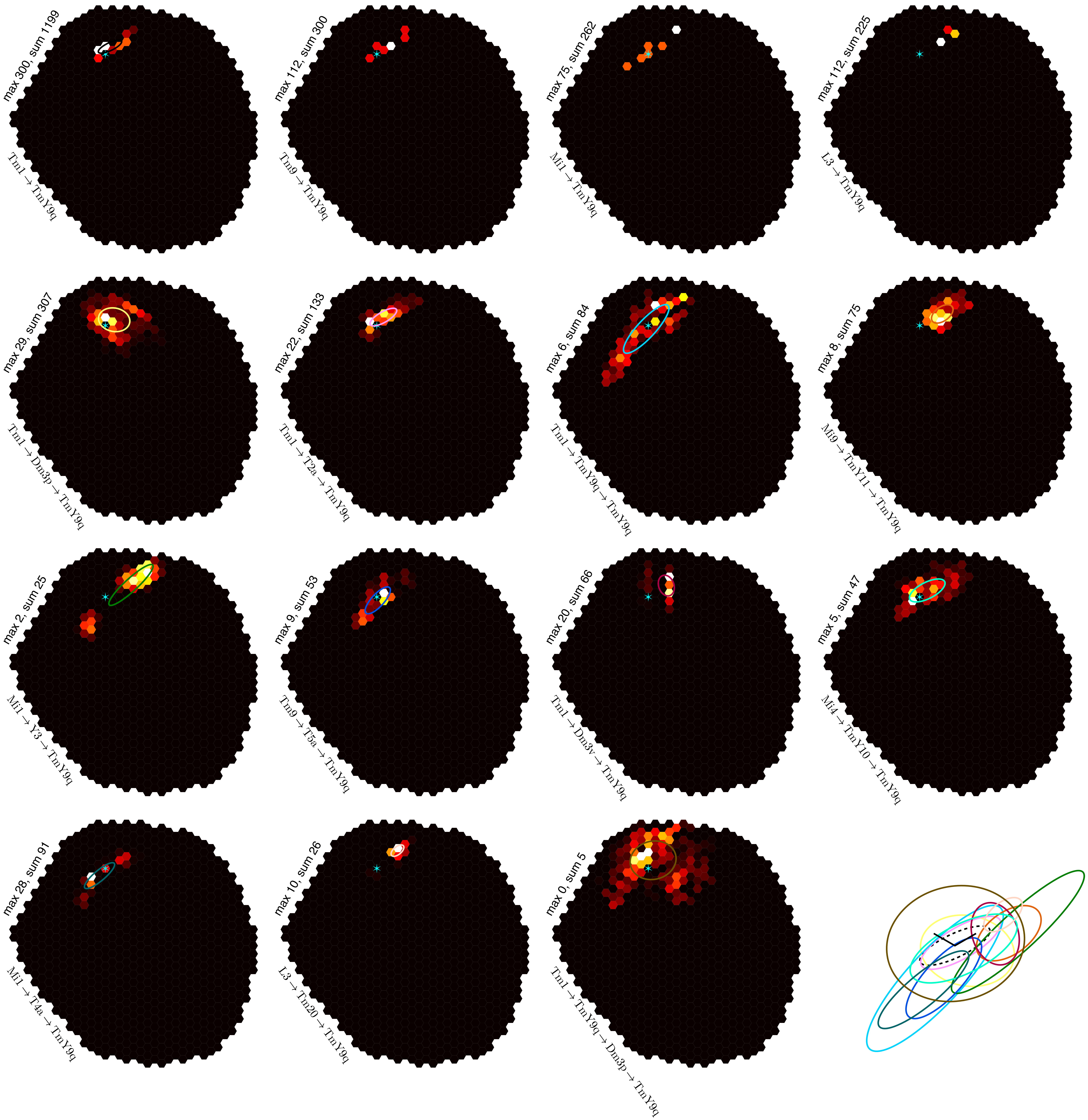
max 300, sum 1199
Tm1 → TmY9q
max 112, sum 300
Tm9 → TmY9q
max 75, sum 262
Mi1 → TmY9q
max 112, sum 225
L3 → TmY9q
max 29, sum 307
Tm1 → Dm3p → TmY9q
max 22, sum 133
Tm1 → T2a → TmY9q
max 6, sum 84
Tm1 → TmY9q → TmY9q
max 8, sum 75
Mi9 → TmY11 → TmY9q
max 2, sum 25
Mi1 → Y3 → TmY9q
max 9, sum 53
Tm9 → T5a → TmY9q
max 20, sum 66
Tm1 → Dm3v → TmY9q
max 5, sum 47
Mi4 → TmY10 → TmY9q
max 28, sum 91
Mi1 → T4a → TmY9q
max 10, sum 26
L3 → Tm20 → TmY9q
max 0, sum 5
Tm1 → TmY9q → Dm3p → TmY9q
